# Congenital Nasal Encephalocele Presenting With Pneumococcal Meningitis and Subsequent Bilateral Sensorineural Hearing Loss: A Case Report of a Pediatric Patient

**DOI:** 10.7759/cureus.115869

**Published:** 2026-09-06

**Authors:** Ajay Bharathan, Nathan Jacob, Cedric V Pritchett, Santino S Cervantes

**Affiliations:** 1 College of Medicine, University of Central Florida College of Medicine, Orlando, USA; 2 Pediatric Otolaryngology, Nemours Children's Hospital, Orlando, USA; 3 Pediatric Surgery, University of Central Florida College of Medicine, Orlando, USA

**Keywords:** bilateral sensorineural hearing loss, cochlear implantation, labyrinthitis ossificans, nasal encephalocele, pediatric meningitis, skull base defect

## Abstract

Congenital nasal encephaloceles are rare anterior skull base defects that may predispose patients to cerebrospinal fluid (CSF) leakage and subsequent meningitis. While sensorineural hearing loss (SNHL) is a known complication of pediatric meningitis, progression from an unrecognized nasal encephalocele to meningitis and bilateral profound hearing loss is infrequently reported. We describe a seven-month-old male who presented with pneumococcal meningitis and status epilepticus requiring intensive care management. Following recovery, he developed severe-to-profound bilateral SNHL. Further evaluation revealed labyrinthitis ossificans and a previously unrecognized nasal encephalocele. The patient underwent bilateral cochlear implantation followed by successful endoscopic repair of the skull base defect. Postoperatively, he demonstrated hearing thresholds within normal ranges and no evidence of recurrence. This case highlights the importance of early recognition of congenital skull base defects in infants presenting with meningitis. It underscores the role of timely imaging and multidisciplinary management in preventing irreversible complications.

## Introduction

Congenital nasal encephaloceles are rare anterior skull base defects characterized by herniation of intracranial contents through the nasofrontal junction, with an estimated incidence ranging from 1 in 5,000 to 1 in 40,000 live births [1]. These lesions may present with a spectrum of clinical findings, including cerebrospinal fluid (CSF) rhinorrhea, nasal obstruction, or a polypoid nasal mass, but are frequently misdiagnosed due to their subtle presentation [2,3]. A major source of morbidity is recurrent or severe bacterial meningitis due to CSF leakage, which provides a direct pathway for bacterial invasion [1,4]. Sensorineural hearing loss (SNHL) occurs in approximately 2.4%-23% of children following meningitis, with higher rates reported in pneumococcal infections [5,6]. Despite this, few reports describe the complete clinical progression from an unrecognized nasal encephalocele to meningitis, subsequent labyrinthitis ossificans, and ultimately bilateral profound hearing loss requiring cochlear implantation.

This case highlights the progression and underscores the importance of early structural evaluation in infants presenting with unexplained meningitis or atypical nasal findings.

## Case presentation

A seven-month-old vaccinated male presented to the emergency department with febrile seizures after being evaluated two days prior for an upper respiratory infection, during which he was positive for fever, rhinorrhea, congestion, decreased appetite, and irritability. He was appropriately vaccinated with two doses of PCV13 and one dose of PCV20 at that time. Lumbar puncture demonstrated hazy, neutrophil-predominant CSF with an elevated protein level of 227 mg/dL and markedly decreased glucose of <2 mg/dL, consistent with bacterial meningitis. CSF cultures subsequently grew Streptococcus pneumoniae, confirming pneumococcal meningitis. Computed tomography of the head with intravenous contrast demonstrated focal hypoattenuation within the left temporal subcortical white matter. The patient was admitted to the pediatric intensive care unit for management of status epilepticus and pneumococcal meningitis.

Treatment included intravenous antibiotics (vancomycin with ceftriaxone) and dexamethasone administration at similar times, antiepileptic medications, and temporary mechanical ventilation. Magnetic resonance imaging (MRI) obtained during admission demonstrated subacute hypoxic-ischemic injury throughout the bilateral cerebral white matter, as well as a chronic left temporal infarct. After a three-week hospitalization, the patient was discharged on antiepileptic therapy with noted global developmental delay but no focal neurologic deficits. Discharge instructions included a request for follow-up with the pediatrician for hearing and visual testing to rule out potential sequelae.

At one month post-discharge (eight months of age), caregivers noted the absence of startle responses to loud auditory stimuli, prompting a formal audiologic evaluation. Initial auditory brainstem response (ABR) testing, conducted at an outside facility before referral, demonstrated severe-to-profound bilateral sensorineural hearing loss. Following otolaryngology evaluation at our institution, repeat in-house ABR confirmed these findings. Otolaryngologic examination revealed normal tympanic membranes but identified a left nasal, purplish, polypoid mass that was somewhat translucent and showed no evidence of CSF leak.

Retrospective review of prior MRI imaging obtained during the original hospitalization revealed a previously unrecognized left nasal cavity mass. Concern for a nasal encephalocele and labyrinthitis ossificans-related hearing loss prompted dedicated anterior skull base imaging with CT sinus in addition to CT temporal bone. Results demonstrated a well-defined, ovoid mass within the anterior left nasal cavity measuring approximately 2.0 x 1.8 x 1.0 cm, with an associated osseous defect of the anterior skull base immediately superior to the lesion, confirming communication with the intracranial compartment. These findings confirmed the diagnosis of a congenital nasal encephalocele (Figure 1). Additionally, CT temporal bone imaging demonstrated bilaterally normally formed and partitioned cochleae with scattered foci of increased attenuation, most notably involving the basal turn on the left and the mid-to-apical turns on the right, consistent with early labyrinthitis ossificans (Figures 2-3).

**Figure 1:**
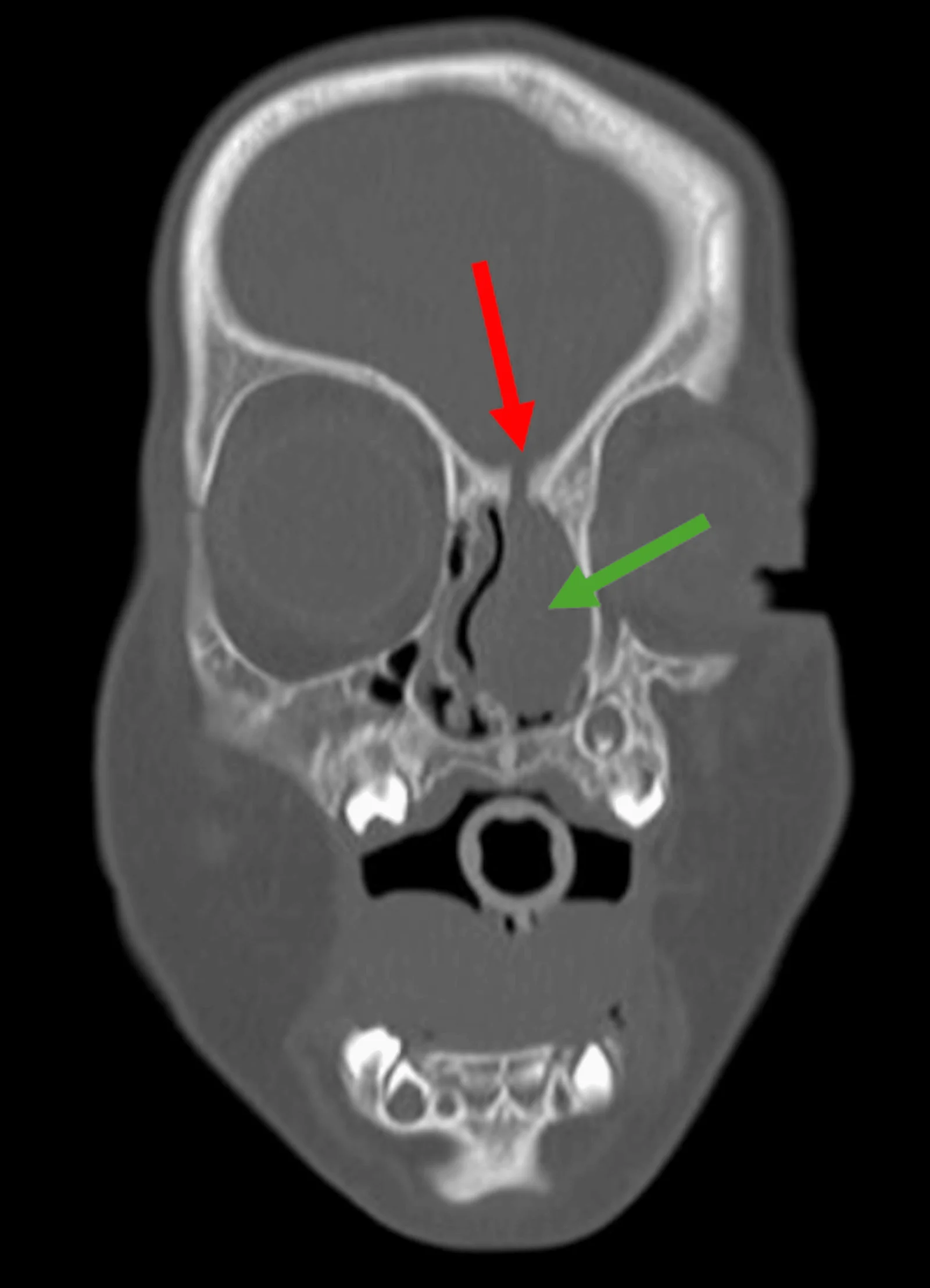
Radiographic image of the patient's skull base defect (red arrow) and nasal encephalocele (green arrow).

**Figure 2:**
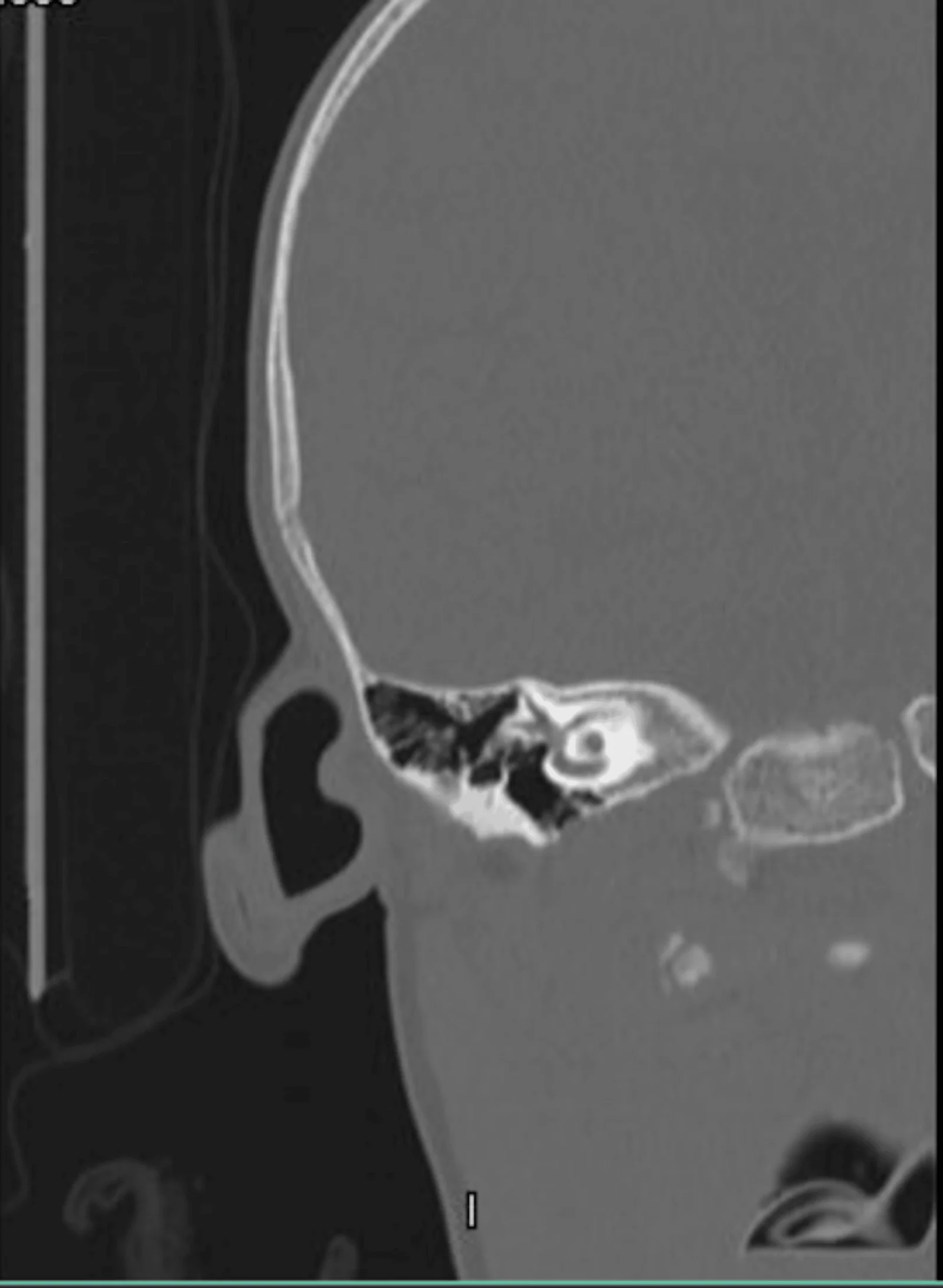
Radiographic imaging of the right cochlea showing scattered foci of increased attenuation involving the mid-to-apical turns.

**Figure 3:**
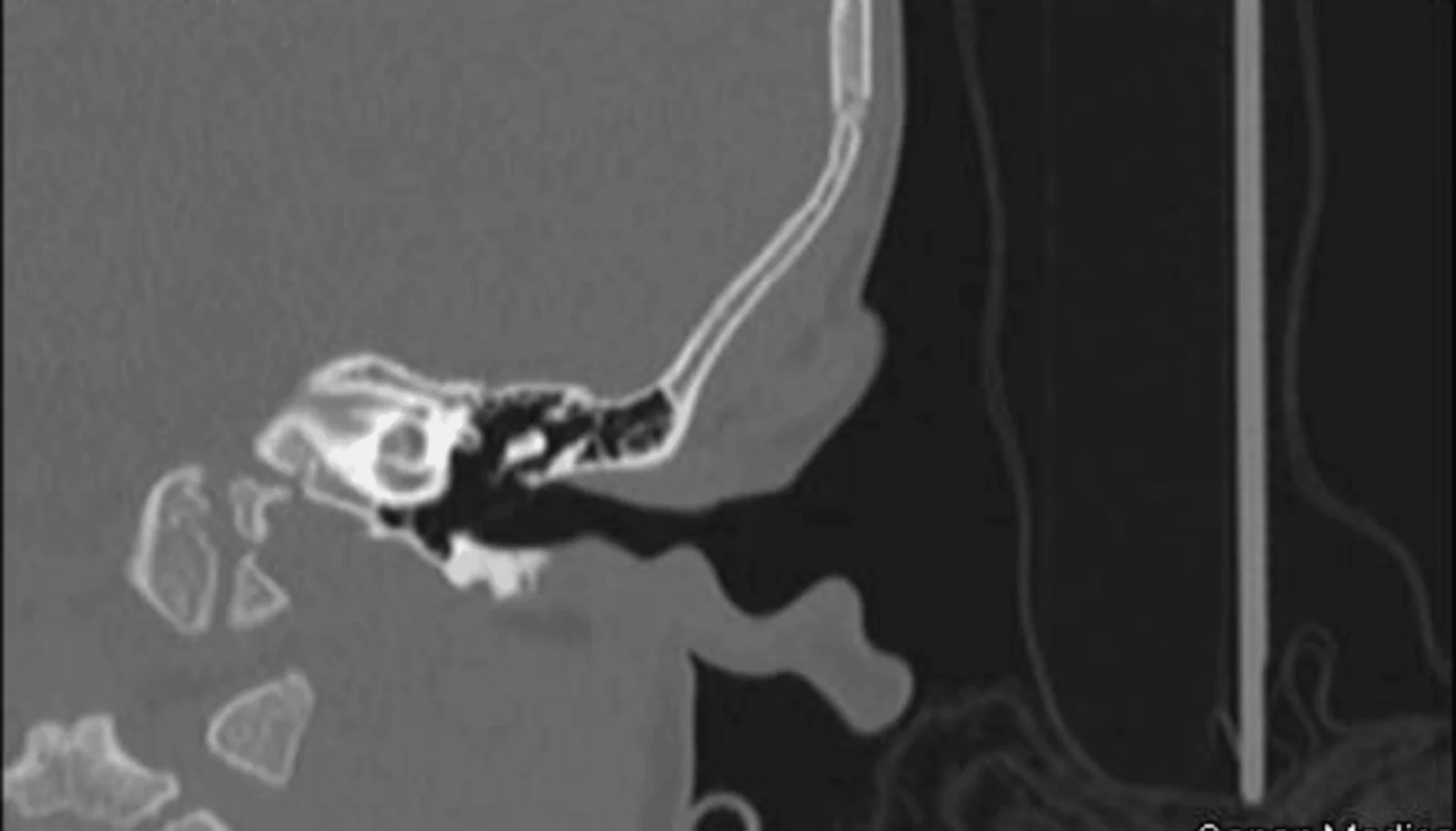
Radiographic image of the left cochlea showing scattered foci of increased attenuation involving the basal turn.

At 11 months of age, the patient underwent simultaneous bilateral cochlear implantation with full electrode insertion (Figure 4). At 13 months, he underwent endoscopic repair of the anterior skull base defect using temporalis fascia and a free mucosal graft. At six months postoperatively, the patient demonstrated cortical responses indicative of sound detection at the primary auditory cortex in the soundfield condition at normal conversational levels while utilizing CI processors. Specifically, P1/P2 complex and N2 responses were present at 70 and 60 dBnHL in both ears. There was also no clinical or radiographic evidence of encephalocele recurrence.

**Figure 4:**
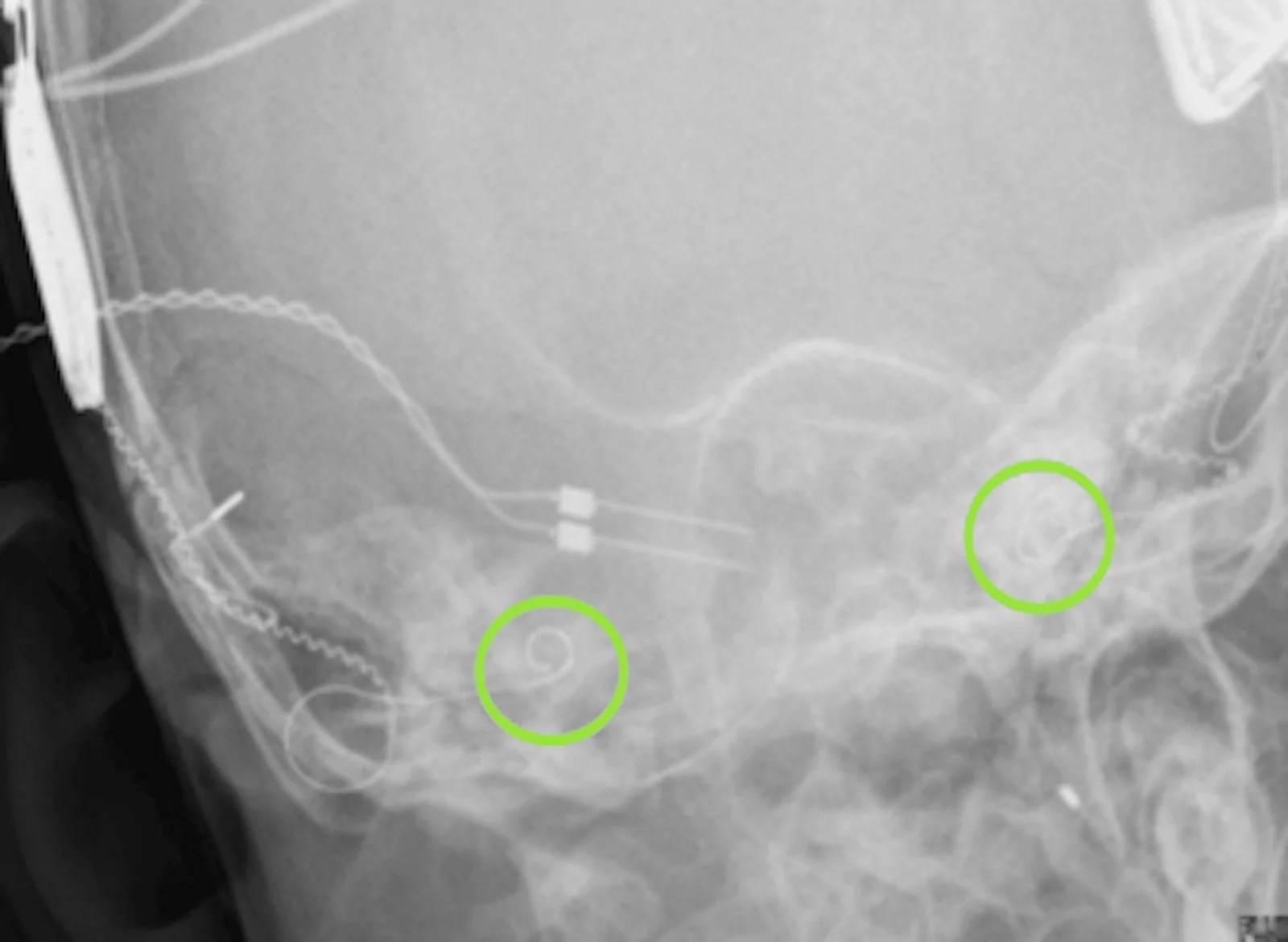
Radiographic image of successful bilateral cochlear implants (green circles) for sensorineural hearing loss.

## Discussion

This case demonstrates the significant morbidity associated with delayed recognition of congenital nasal encephaloceles. Although the association between anterior skull base defects and meningitis is well established, these lesions are frequently underdiagnosed due to their subtle clinical presentation and potential to mimic benign nasal polyps or other congenital masses [1-4]. CSF leakage associated with encephaloceles creates a direct conduit for bacterial entry into the intracranial space, predisposing patients to severe or recurrent meningitis. In this patient, delayed identification of the underlying defect resulted in the rapid progression of pneumococcal meningitis to subsequent bilateral SNHL due to labyrinthitis ossificans, an irreversible complication that may begin within weeks following infection [7,8]. Bacterial meningitis, particularly when caused by Streptococcus pneumoniae, remains a leading cause of acquired sensorineural hearing loss in children, with hearing loss occurring in up to 22%-30% of survivors in some series [5].

This case highlights a clinically important progression that is infrequently documented: (1) congenital skull base defect, (2) meningitis, (3) cochlear ossification, and (4) profound hearing loss. Recognition of this cascade is critical, as earlier identification of the encephalocele may have prevented the infectious complication and its sequelae. These findings support the need for early imaging in infants presenting with unexplained meningitis, persistent rhinorrhea, nasal obstruction, or nasal masses of unclear origin. High-resolution CT and MRI are complementary modalities, with combined imaging achieving high diagnostic sensitivity for identifying skull base defects [4,9].

Cochlear implantation remains the standard of care for post-meningitis deafness with profound SNHL [9]. In the case of bacterial meningitis, early audiometric evaluation and labyrinthine imaging are critical, as delayed detection occurs in up to 25% of cases and may adversely impact rehabilitation outcomes due to progressive cochlear ossification [10]. Rapid progression to labyrinthitis ossificans can limit cochlear implant candidacy due to ossification of the cochlea, as this may limit electrode insertion if intervention is delayed [8]. In this context, early cochlear implantation, often within months of diagnosis, has been associated with improved audiologic outcomes compared to delayed intervention [9]. While implantation in the setting of cochlear ossification may present technical challenges and reduced speech outcomes, successful auditory rehabilitation is still achievable [11,12].

Surgical management of anterior skull base defects can be accomplished by traditional craniotomy or endoscopic approaches. Endoscopic repair of anterior skull base defects has become the preferred approach in pediatric populations due to reduced morbidity and improved visualization compared to open approaches [13,14]. Endoscopic techniques typically involve either free grafting or pedicled (vascularized) grafting. Free grafts, such as fascia or fat, rely on surrounding tissue for revascularization, whereas pedicled grafts maintain an intact blood supply, enhancing healing and durability of the reconstruction. Success rates exceeding 95% have been reported in experienced centers [15]. Although younger age may be associated with higher rates of postoperative CSF leak or recurrence, successful repairs have been performed even in very young infants when clinically indicated [3,14].

## Conclusions

Congenital nasal encephaloceles, although rare, can result in severe complications if not identified early. This case illustrates progression from an unrecognized skull base defect to pneumococcal meningitis, bilateral sensorineural hearing loss, and the need for cochlear implantation. Early neuroimaging should be considered in infants presenting with unexplained meningitis or atypical nasal findings. Prompt diagnosis and multidisciplinary management are essential to prevent irreversible infectious and neurologic complications.
